# Occurrence and genetic characterization of *Giardia duodenalis* and *Cryptosporidium* spp. from adult goats in Sichuan Province, China

**DOI:** 10.1371/journal.pone.0199325

**Published:** 2018-06-18

**Authors:** Zhijun Zhong, Rui Tu, Hongping Ou, Guangwen Yan, Jiaming Dan, Qicheng Xiao, Ya Wang, Suizhong Cao, Liuhong Shen, Junliang Deng, Zhicai Zuo, Xiaoping Ma, Ziyao Zhou, Haifeng Liu, Shumin Yu, Zhihua Ren, Yanchun Hu, Guangneng Peng

**Affiliations:** 1 Key Laboratory of Animal Disease and Human Health of Sichuan Province, College of Veterinary Medicine, Sichuan Agricultural University, Sichuan, P.R. China; 2 Chengdu Agricultural College, Sichuan, P.R. China; 3 College of Animal Science, Xichang University, Xichang, P.R. China; University of Minnesota, UNITED STATES

## Abstract

*Giardia duodenalis* and *Cryptosporidium* spp. are common gastrointestinal protozoa in mammals. Many studies have been conducted on the distribution of *G*. *duodenalis* and *Cryptosporidium* spp. genotypes in sheep and cattle. However, in China, information about molecular characterization and genetic analysis of *G*. *duodenalis* and *Cryptosporidium* spp. in goats is limited. In this study, 342 fecal samples from adult goats were collected from 12 farms in Sichuan Province, China. The occurrence of *G*. *duodenalis* and *Cryptosporidium* spp. in adult goats was 14.9% (51/342) and 4.7% (16/342), respectively. All *G*. *duodenalis* were identified as assemblage E, with two novel genotypes (assemblages E17 and E18) being detected at the beta-giardin (*bg*) locus. Based on three loci—beta-giardin (*bg*), triose phosphate isomerase (*tpi*), and glutamate dehydrogenase (*gdh*)—multilocus sequence typing revealed three novel multilocus genotypes (MLGs) of assemblage E (MLG-E1, E2, E3 (sc)). Small Subunit (SSU) rRNA-based PCR identified two *Cryptosporidium* species, namely *C*. *xiaoi* (11/16) and *C*. *suis* (5/16). This study is not only the first to report *C*. *suis* infection in adult goats in China but is also the first to use the MLG approach to identify *G*. *duodenalis* in adult goats.

## Introduction

*Giardia duodenalis* and *Cryptosporidium* spp. are two genera of intestinal parasitic protozoa that infect humans and a broad range of animals, including livestock, companion animals, and wildlife [1–3]. Infection is acquired following ingestion of highly resilient, infective stages (cysts or oocysts) via the fecal-oral route [4]. Damage to animals is dependent on multiple factors, such as the strain of the parasite involved and the immunological and nutritional status of the host, and clinical manifestations vary from asymptomatic to acute or chronic diarrheal disease [5,6].

*Giardia duodenalis* and *Cryptosporidium* spp. are two of the most common identified parasitic protists in ruminants (i.e., cattle, goat, and sheep) and *G*. *duodenalis* is considered a multispecies complex with at least eight distinct assemblages (A–H). It has been reported that *G*. *duodenalis* assemblages A, B, and E are capable of infecting goats [7], and of these, assemblage E has been commonly detected in goats worldwide [8–11]. The zoonotic assemblages A and B have also been detected in goats, which suggests a potential threat to human beings [12–16].

In recent years, *G*. *duodenalis* has been identified in a wide variety of animals, including cattle (assemblages A, B, and E), sheep (assemblages A, B, and E), goats (assemblages A, B, and E), dogs (assemblages A, C and D), cats (assemblages A, F), pigs (assemblages A, B, D, E, and F), rabbits (assemblages B and E), rodents (assemblages A, B, and G), non-human primates (NHPs) (assemblages A, B, and E), and some other wild animals in China [17]. Of the abovementioned animals, information about production animals, including cattle and sheep, is more frequently reported, mostly in provinces with developed husbandry. In China, *G*. *duodenalis* infection in goats is limited with reports from only four provinces (Anhui, Henan, Shananxi, and Heilongjiang) [11,13,14]. Similarly, *Cryptosporidium* spp. has also been widely reported in various animals in China, especially for ruminants, such as cattle, sheep, and goats [18]. Several *Cryptosporidium* species (*C*. *ubiquitum*, *C*. *xiaoi*, *C*. *parvum*, *C*. *hominis*, *C*. *andersoni*, and rat genotype II) have been reported in goats worldwide with *C*. *hominis* and *C*. *parvum* frequently reported in humans, especially in developing countries including China, which suggests the potential occurrence of zoonotic transmission between goats and humans [18]. However, in China, only *C*. *ubiquitum*, *C*. *xiaoi*, *C*. *parvum*, and *C*. *andersoni* have been identified in goats, with *C*. *ubiquitum* and *C*. *xiaoi* being predominant [7,14,19,20]. The *Cryptosporidium* spp. genotypes that have the potential to infect goats are still not clear.

Recent studies have mostly focused on reservoirs of *G*. *duodenalis* and *Cryptosporidium* spp. in cattle, particularly calves, and only a limited number of reports have focused on goats as the reservoir for these two parasites [21]. According to the National Bureau of Statistics of the People’s Republic of China, in 2016, the total population (~15.67 million heads) of goats in Sichuan Province was the fourth largest in China, which has the largest goat population in the world [5]. However, no information about *G*. *duodenalis* or *Cryptosporidium* spp. infection in goats is available in Sichuan Province. To gain a better understanding about the genetic diversity and frequency of *G*. *duodenalis* and *Cryptosporidium* species in adult goats, we conducted the first known molecular study on *G*. *duodenalis* and *Cryptosporidium* spp. infection for Sichuan Province, China. Here, we report for the first time the occurrence and genetic characterization of *G*. *duodenalis* infection in adult goats in Sichuan Province, China.

## Methods

### Ethics statement

This study was reviewed and approved by the Institutional Animal Care and Use Committee of Sichuan Agricultural University under permit number DYY-S20174604. The field studies did not involve any endangered or protected animal species. Prior to the study, permission was granted by the owners of 12 farms. No other specific permits were required for the described field studies. In this study, all fecal samples were carefully collected cautiously from the rectum of each goat without causing discomfort.

### Sample collection

**Fig 1:**
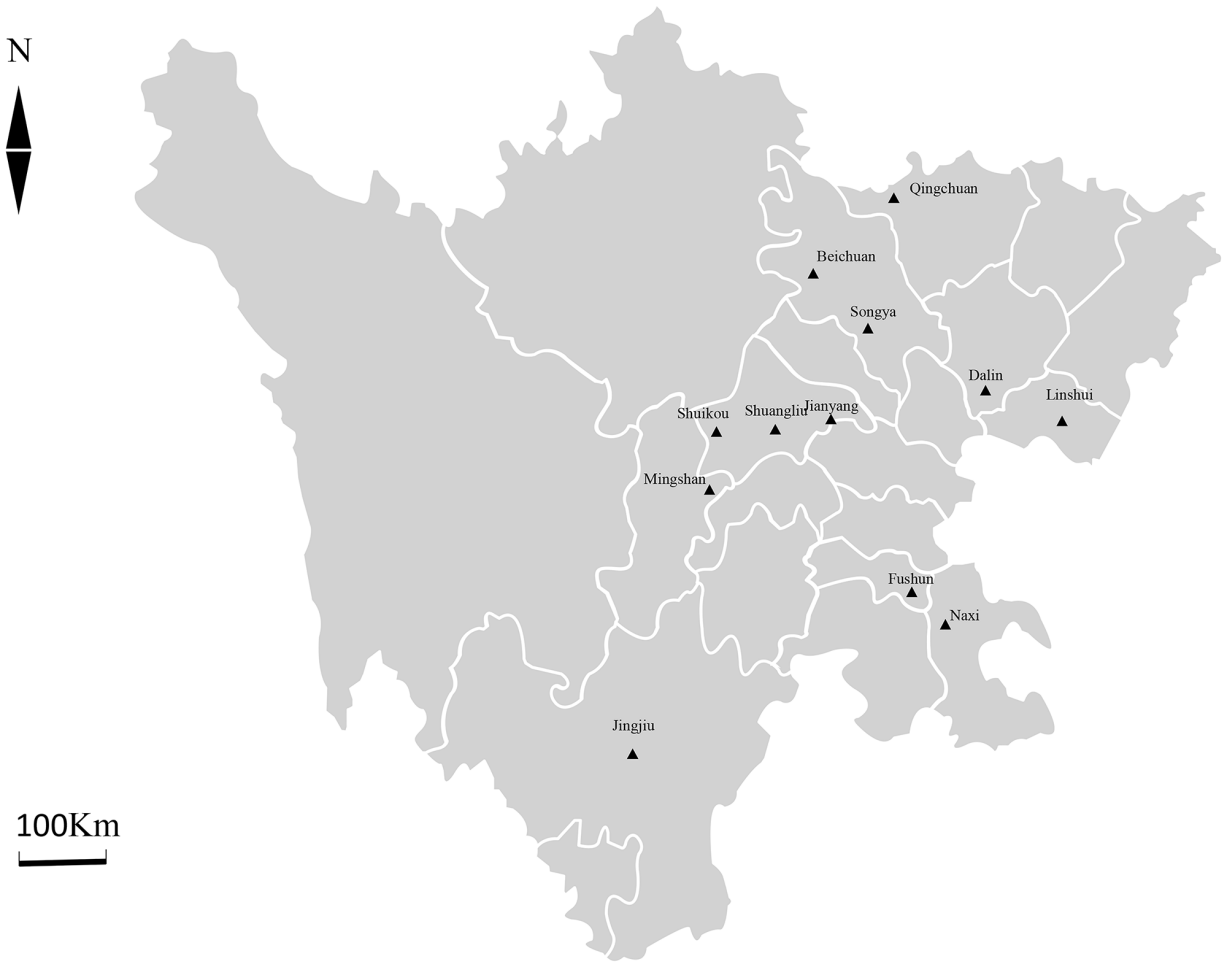
Sampling sites (filled triangles) in Sichuan Province, China.

Sichuan Province, located in southwestern China, has a subtropical monsoon climate, where the breeding industry is still in the initial development stage although it has a long history of traditional husbandry. From June to August 2017, a total of 342 fecal samples were collected from goats at 12 different farms in Sichuan Province, China, with a history of animals with diarrhea, including Qingchuan (105°50′E, 32°26′N), Beichuan (104°18′E, 31°53′N), Songya (104°50′E, 31°22′N), Dalin (106°06′E, 30°50′N), Linshui (106°37′E, 30°27′N), Shuikou (103°27′E, 30°24′N), Shuangliu (104°03′E, 30°34′N), Jianyang (104°32′E, 30°24′N), Mingshan (103°02′E, 30°0′N), Fushun (104°46′E, 29°20′N), Naxi (105°26′E, 28°52′N), and Jingjiu (102°20′E, 27°50′N) (Fig 1). The city-level map was provided by the National Geomatics Centre of China (National Geomatics Centre of China, Beijing, China, http://ngcc.sbsm.gov.cn/).

The 12 farms are distributed throughout Sichuan Province, except the western region where animal husbandry is relatively undeveloped. Of the 12 farms, six (Qingchuan, Dalin, Beichuan, Jianyang, Fushun, and Jingjiu) practiced intensive feeding, while the other six farms are free-range. All goats included in the study were more than 1 year old. Feces were collected directly from the rectum of each goat into a 50-mL centrifuge tube, which was immediately capped, labeled, and placed in a box with ice. Fecal samples were transported to the Sichuan Agricultural University and stored in 2.5% potassium dichromate at 4 °C. All samples were processed within 24 h after collection.

### DNA extraction and PCR amplification

Before DNA extraction, feces were washed with distilled water to remove potassium dichromate. DNA was extracted from the feces using a PowerSoil^®^ DNA isolation kit (MoBio, Carlsbad CA, USA) according to the manufacturer`s instructions and stored at −20°C in 100 μL of the solution buffer provided in the kit [22].

For detection of *Giardia* spp., DNA samples were subjected to nested PCR amplifications based on the detection of beta-giardin (*bg*), triose phosphate isomerase (*tpi*), and glutamate dehydrogenase (*gdh*) as previously described [23]. Nested PCR of *Cryptosporidium* spp. was performed targeting the small subunit (SSU) rRNA gene [24]. The primers and amplification conditions used in this study (Table 1) have been previously described [23, 24]. The PCR reaction was composed of 12.5 μL 2× Taq PCR Master Mix (KT201-02, Tiangen, Beijing, China), 8.5 μL deionized water (Tiangen, Beijing, China), 2 μL DNA, and 1 μL each of set primers, for a total volume of 25 μL. Positive and negative controls were included in each test. Secondary PCR products were visualized by 1% agarose gel electrophoresis and staining with Golden View.

**Table 1:** Primer sequences, annealing temperatures and the fragment lengths of the genes used in this study.

Gene	Primer	Sequence(5’-3’)	Annealing Temperature(°C)	Fragment Length(bp)	Reference
*bg*	F1	AAGCCCGACGACCTCACCCGCAGTGC	60	530	[23]
	R1	GAGGCCGCCCTGGATCTTCGAGACGAC			
	F2	GAACGAACGAGATCGAGGTCCG	55		
	R2	CTCGACGAGCTTCGTGTT			
*tpi*	F1	AAATIATGCCTGCTCGTCG	50	530	[23]
	R1	CAAACCTTITCCGCAAACC			
	F2	CCCTTCATCGGIGGTAACTT	50		
	R2	GTGGCCACCACICCCGTGCC			
*gdh*	F1	TTCCGTRTYCAGTACAACTC	50	511	[23]
	R1	ACCTCGTTCTGRGTGGCGCA			
	F2	ATGACYGAGCTYCAGAGGCACGT	50		
	R2	GTGGCGCARGGCATGATGCA			
18S	F1	TTCTAGAGCTAATACATGCG	55	800	[24]
	R1	CCCTAATCCTTCGAAACAGGA			
	F2	GGAAGGGTTGTATTTATTAGATAAAG	58		
	R2	AAGGAGTAAGGAACAACCTCCA			

### Sequencing and phylogenetic analysis

The secondary PCR products were sent to Invitrogen (Shanghai, China) and sequenced in both directions. To determine the species/assemblages of *Giardia* and *Cryptosporidium* species/genotypes, sequences were aligned with reference sequences from GenBank using BLAST (http://blast.ncbi.nlm.nih.gov) and Clustal X. For the phylogenetic analysis, sequences at the three loci obtained in this study were concatenated to form one multilocus sequence for each isolate. A neighbor-joining tree was constructed using Mega 5 (http://www.megasoftware.net/) based on the evolutionary 124 distances calculated using the Kimura 2-parameter model with 1000 replications for the bootstrap analysis.

### Nucleotide sequence accession numbers

Representative nucleotide sequences were deposited into the GenBank database under the following accession numbers: MG602956–MG602963. GenBank accession numbers of reference sequences are listed as follows: E5 (KY769092) and E8 (KY633465) for the *bg* loci; E2 (KT92262) and E3 (KT92259) for the *tpi* loci; and E3 (KF843925) and E4 (KF843926) for the *gdh* loci.

## Results and discussion

### Giardia duodenalis

*Giardia duodenalis* infection in goats has been reported from many countries such as Ghana, Malaysia, India, Greece, Tanzania, Iran, Spain, the Netherlands, and China [8–10,12,13,15,16,25,26]. However, in China, information regarding *G*. *duodenalis* infection in goats is limited, with only three reports available [11,13,14]. Our study, for the first time, reports the occurrence and genetic characterization of *G*. *duodenalis* infection in adult goats in Sichuan Province, China.

In the present study, goats from 10 of the 12 farms showed presence of *G*. *duodenalis*, with the positive rate ranging from 5–37.5% (Table 2). The highest occurrence (37.5%, 15/40) was found in Linshui Farm which is free-ranging and relatively poorly managed. *Giardia duodenalis* was detected in 51 of 324 (14.9%) fecal samples from adult goats, which was higher than that observed in Shaanxi (10.8%) [14], Anhui (6.3%) [11], or Heilingjiang (2.9%) [13], but lower than that in Henan (17.3%) [14]. Moreover, only two studies related to goat kids are available to date, but the infection rates of *G*. *duodenalis* in kids in Greece (40.4%) [9] and the Canary Islands (42.2%) in Spain [15] are significantly higher than what has been reported in any study for adult goats. This suggests that *G*. *duodenalis*-infection may be associated with age, which had been previously proved in sheep and cattle [27,28]. However, whether this hypothesis is reliable still requires confirmation with a more extensive epidemiological investigation of goats of various ages.

**Table 2:** Occurrence of *C*. *xiaoi*, *C*. *suis* and *G*. *duodenalis* in adult goats in Sichuan Province, China.

Farm	No. tested	*Cryptosporidium*		*Cryptosporidium* No.(%) of positive	*G*.*duodenalis*	*G*. *duodenalis* No.(%) of positive
		*C*.*xiaoi*	*C*.*suis*			
Mingshan^a^	41	6		14.6 (95% CI*:3.3~25.9)	9	22.0 (95% CI:8.7~35.2)
Naxi^a^	26		5	19.2 (95% CI:0.5~31.4)	2	7.7 (95% CI:-3.3 ~18.6)
Shuikou^a^	40				2	5.0 (95% CI:-2.1~12.1)
Songya^a^	24				0	0
Linshui^a^	40	1		2.5 (95% CI:-2.5~7.5)	15	37.5 (95% CI:12.1~57.9)
Shuangliu^a^	25	4		16.0 (95% CI:0.5~31.4)	5	20.0 (95% CI:3.1~36.8)
Jingjiu^b^	36				3	8.3 (95% CI:-1.1~17.8)
Qingchuan^b^	21				6	28.6 (95% CI:7.5~49.6)
Dalin^b^	26				0	0
Beichuan^b^	23				1	4.3 (95% CI:-4.7~13.4)
Jianyang^b^	20				1	5.0 (95% CI:-5.5~15.5)
Fushun^b^	20				7	35.0 (95% CI:12.1~57.9)
Total	342	11	5	4.7 (95% CI:2.4%~6.9)	51	14.9 (95% CI:11.1~18.7)

*CI: confidence intervals

^a^: free-ranging

^b^: intensive farming

The genetic diversity of *G*. *duodenalis* was analyzed by sequencing the *bg*, *tpi*, and *gdh* loci with 51, 37, and 33 sequences were obtained, respectively (Table 3). Sequence analysis of 51 *G*. *duodenalis* positive samples showed mono-infection with the ruminant-specific assemblage E at the three loci, which is similar to the results of previous investigations on adult goats [8,10,11,29]. According to previous studies, the zoonotic genotypes A and B have been detected in goats, which suggests a zoonotic threat to human beings [12]. In the present study, our findings indicated that adult goats may not be a potential reservoir for zoonotic genotypes of *G*. *duodenalis* in Sichuan Province. However, recently, scientists concluded that assemblage E should also be considered potentially zoonotic [7,30]. More epidemiological research is needed regarding the potentially zoonotic genotypes of assemblage E.

**Table 3:** Multilocus characterization of *Giardia duodenalis* isolates in adult goats based on the *bg*, *tpi* and *gdh* genes.

Isolate	Farm	Subtype			
		*bg*	*tpi*	*gdh*	MLG type
BC07	Beichuan	-	E3	-	-
CD02,06,08,22	Shuangliu	E5	E2	E4	MLG1(sc)
CD01		E5	E2	-	-
GA03,17,19	Linshui	E5	E2	E4	MLG1(sc)
GA06,18		E5	-	E4	-
GA05		E5	-	-	-
GY17,18,20,26,27,29,35,37	Qingchuan	E5	E2	E4	MLG1(sc)
GY05,08,23,28,40		E5	E2	-	-
GY25,32		E5	-	-	-
JY13	Jianyang	E8	E3	E3	MLG2(sc)
LZ012,15	Naxi	E5	E2	E4	MLG1(sc)
QL27	Shuikou	E5	E2	E4	MLG1(sc)
QL08		E5	E2	-	-
XCA09	Jingjiu	E17^#^	E2	E4	MLG3(sc)
XCA21		E5	E2	-	-
XCA23		E5	-	-	-
YAA15,26,29,37,38,39	Mingshan	E5	E2	E4	MLG1(sc)
YAA21		E5	-	-	-
YAA18,36		E5	E2	-	-
ZG01,03,06,12,17	Fushun	E5	E2	E4	MLG1(sc)
ZG16		E18^#^	E2	-	-
ZG14		E5	-	-	-

“-” represents PCR negative.

“^#^” represents the novel genotypes.

“sc” represents Sichuan Province.

*Bg* locus sequences exhibited an unexpectedly high occurrence of assemblage E5 (94.1%), with only one isolate (JY13) identified as E8 (2.0%). Two novel subtypes, named E17 (2.0%) and E18 (2.0%), were also detected. Among the 43 *tpi* sequences, no genetic variation was observed, with assemblage E2 (97.7%) being predominant and only one isolate (BC07) from Beichuan identified as assemblage E3 (2.3%). At the *gdh* locus, only two known subtype assemblages, E4 (97.0%) and E3 (3.0%), were found among the 33 sequences, of which the majority of the isolates were identified as assemblage E4, except one isolate from Jianyang (JY13) was identified as assemblage E3. Among the *bg*, *tpi*, and *gdh* loci, predominant subtypes of assemblages E at each locus (E5 (94.1%) at the *bg* locus, E2 (97.7%) at the *tpi* locus, and E4 (97.0%) at the *gdh* locus) were also commonly detected in sheep, cattle, yaks, and pigs, demonstrating a potential risk of cross-species transmission of *G*. *duodenalis* to different animals [27,29,31,32].

**Fig 2:**
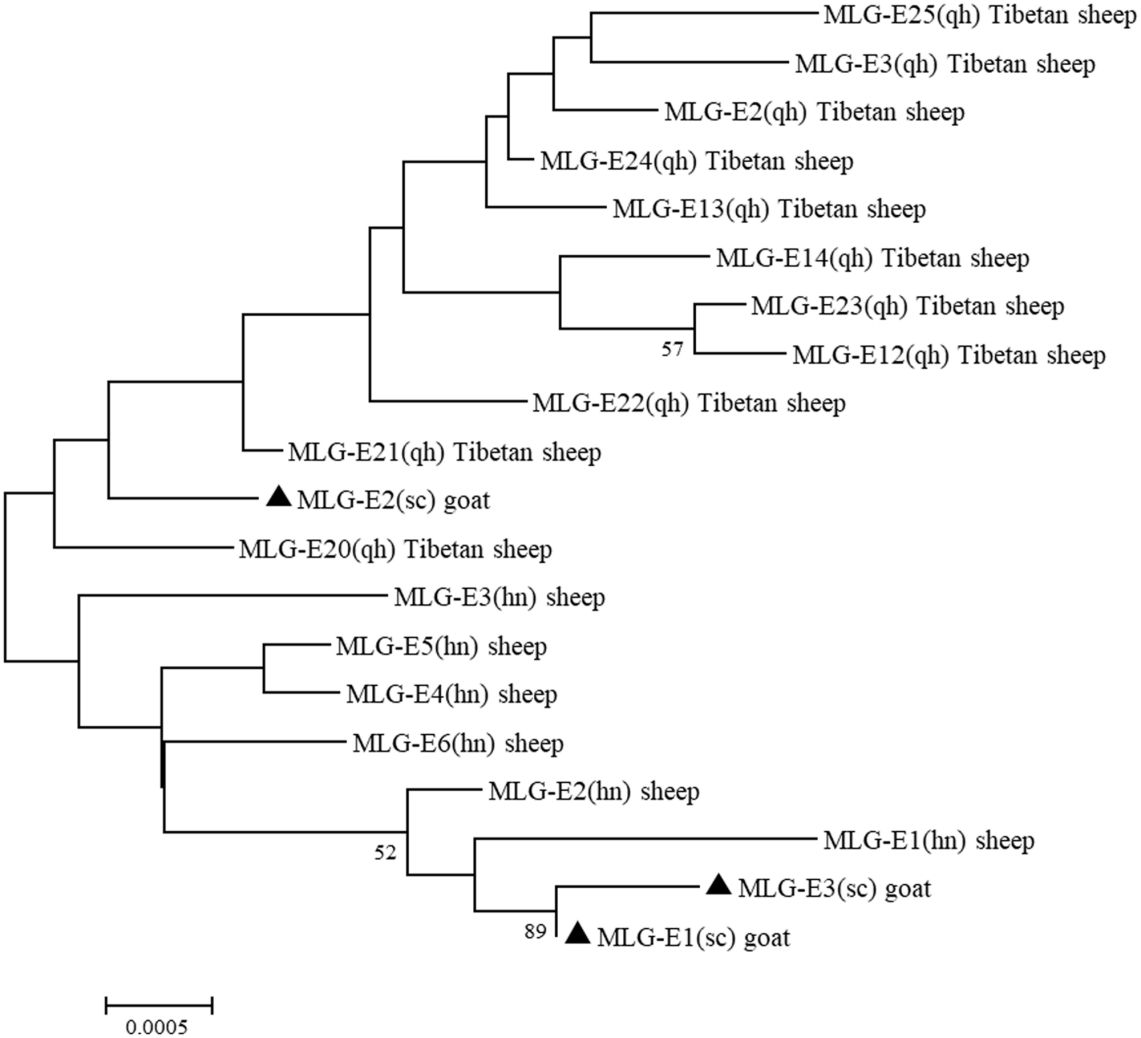
Phylogenetic relationships of *Giardia duodenalis* multilocus genotypes (MLGs). The neighbor-joining tree was constructed using concatenated sequences of the *bg*, *gdh*, and *tpi* genes. Bootstrap values greater than 50% from 1000 replicates are shown. “sc”, “qh” and “hn” represent Sichuan, Qinghai, and Henan, respectively.

Furthermore, using multilocus sequence typing, we analyzed 31 positive isolates that were successfully amplified on all the three loci (*bg*, *tpi*, and *gdh)*, forming three novel assemblage E MLGs (MLG-E1 (sc), MLG-E2 (sc), and MLG-E3 (sc)) (Table 2). Assemblage E MLGs were only identified from nine farms. MLG-1 (sc) was observed in seven farms while MLG-2 (sc) (3.2%, 1/31) and MLG-E3 (sc) (3.2%, 1/31) were found only at the Jiangyang and Jingjiu farms, respectively, suggesting MLG-1 (sc) (93.5%, 29/31) was the predominant MLG (sc), while MLG-2 (sc) and MLG-E3 (sc) may be farm-unique. Compared with other MLGs detected in previous studies, lower genetic heterogeneity was observed (only 3 MLGs detected) in *G*. *duodenalis*, which was mainly reflected by the limited subtype diversity of assemblage E with only 4, 2, and 2 subtypes yielded at the *bg*, *gdh*, and *tpi* loci, respectively [29,33]. Phylogenetic analysis indicated that the three novel assemblage E MLGs were genetically distinct from those isolated from sheep in Qinghai and Henan Provinces, China [29,31]. Moreover, MLG-E1 (sc) and MLG-E3 (sc) were placed in the major cluster of MLGs from Tibetan sheep in Qinghai, whereas MLG-E2 (sc) was clustered with MLGs from sheep in Henan (Fig 2). This result revealed the presence of host-specific clusters of MLGs from adult goats in Sichuan Province.

In short, our study, for the first time, reports the occurrence and genetic characterization of *G*. *duodenalis* infection in adult goats in Sichuan Province, China. This first-time observation requires a more extensive epidemiological investigation in goats of various ages.

### *Cryptosporidium* spp.

In the present study, goats from 4 of the 12 farms contained *Cryptosporidium* spp., with the positive rate ranging from 2.5% to 19.2% (Table 1). None of the *Cryptosporidium*-positive farms practiced intensive farming, which suggests the occurrence of *Cryptosporidium* spp. in goats may be closely related to management of the farms. The overall occurrence of *Cryptosporidium* spp. in this study was 4.7% (16/342, Table 1), which was higher than that observed in Henan (2.8%, 28/1017) [20] and close to that in Chongqing (6.5%, 16/248) [20] and Guangdong (5.5%, 5/91) [19]. However, it was much lower than the positive rate in Shaanxi (11.3%, 55/485), Henan (34.0%, 49/144), Shandong (18.0%, 18/100), Hubei (11.7%, 13/111) and Shanghai (10.9%, 33/302) [14,19].

Among the 16 positive samples, two *Cryptosporidium* species (*C*. *xiaoi* and *C*. *suis*) were identified. *C*. *xiaoi* was the most predominant genotype detected in three farms, while *C*. *suis* was only found in Naxi. Todate, this is the first molecular identification of *C*. *suis* infection in adult goats. *C*. *xiaoi* was the predominant species found in this study (68.8%, 11/16), which is consistent with that reported in previous surveys in China [14,19]. However, it is different from studies in Henan and Chongqing [20], which revealed that *C*. *andersoni* and *C*. *ubiquitum* are predominant species, respectively. Other studies found that *C*. *parvum* was the dominant species in goats [34–36]. In this study, *C*. *parvum*, *C*. *andersoni*, and *C*. *ubiquitum*, which were considered zoonotic subtypes, were not detected. The difference in genotype between our survey and previous studies may be related to host age, raising density, geographical ecological conditions, and examination methods [7, 14, 19,20].

## Conclusions

This is the first known report on the occurrence and genetic characterizations of *G*. *duodenalis* and *Cryptosporidium* spp. in adult goats in Sichuan Province, China. We found that assemblage E of *G*. *duodenalis* was dominant in adult goats in this region. For the first time, we used an MLG approach to identify *G*. *duodenalis* in adult goats, and we detect three novel assemblage E MLGs. For *Cryptosporidium* spp., two *Cryptosporidium* species (*C*. *xiaoi* and *C*. *suis*) were observed, which is, notably, the first time *C*. *suis* infection has been identified in adult goats. For a better understanding of the epidemiology and genotypes of *G*. *duodenalis* and *Cryptosporidium* spp. in goats, further investigation with a larger sample of goats of different ages is needed.
